# Common *MC1R* Variants and Parkinson Disease Progression

**DOI:** 10.1001/jamaneurol.2026.2998

**Published:** 2026-09-08

**Authors:** Jackson G. Schumacher, Xinyuan Zhang, Jian Wang, Johannes M. Dijkstra, Hirohisa Watanabe, Xiang Gao, Marianna Cortese, Eric A. Macklin, Michael A. Schwarzschild, Xiqun Chen

**Affiliations:** 1 Department of Neurology, Massachusetts General Hospital, Harvard Medical School, Boston; 2 Aligning Science Across Parkinson’s Collaborative Research Network, Chevy Chase, Maryland; 3 Channing Division of Network Medicine, Brigham and Women’s Hospital, Harvard Medical School, Boston, Massachusetts; 4 Department of Biostatistics, The University of Texas MD Anderson Cancer Center, Houston; 5 Department of Neurology, School of Medicine, Fujita Health University, Toyoake, Japan; 6 Department of Nutritional Sciences, The Pennsylvania State University, University Park; 7 Departments of Epidemiology and Nutrition, Harvard T. H. Chan School of Public Health, Boston, Massachusetts; 8 Now with Department of Nutrition and Food Hygiene, School of Public Health, Institute of Nutrition, Fudan University, Shanghai, China

## Abstract

**Importance:**

Melanocortin 1 receptor (*MC1R*) is a key regulator of pigmentation and oxidative stress implicated in Parkinson disease (PD). *MC1R* loss-of-function variants, defined by experimentally demonstrated reductions in *MC1R* function, are carried by more than 60% of individuals of European descent.

**Objective:**

To determine whether *MC1R* loss-of-function variants are associated with accelerated PD progression.

**Design, Setting, and Participants:**

This longitudinal cohort study used data from July 2010 to January 2026 in the Parkinson Progression Markers Initiative (PPMI) cohort and June 2009 to June 2019 in a replication cohort using 3 US-based multicenter randomized clinical trials (SURE-PD phase 2, SURE-PD3, and STEADY-PD III), with up to 12 years of follow-up. Analysis was performed in May 2026. Of 926 PPMI participants with PD and available genomewide sequencing data, 66 without dopamine deficiency and 51 with pathogenic variants in known PD-associated genes were excluded. The remaining 809 participants were stratified by *MC1R* loss-of-function carrier status (505 carriers and 304 noncarriers) and classified as having sporadic PD (n = 383) or monogenic PD (n = 426) based on *LRRK2* and *GBA* carrier status. The replication cohort included 587 participants with PD (410 carriers and 177 noncarriers). An additional 53 PPMI participants with prodromal PD (34 carriers and 19 noncarriers) were assessed.

**Exposures:**

*MC1R* loss-of-function carrier status.

**Main Outcomes and Measures:**

Rate of motor decline per Movement Disorder Society Unified Parkinson’s Disease Rating Scale Part III score, assessed via linear mixed-effects models adjusted for age at onset, sex, race, baseline score, and levodopa equivalent daily dose. Phenoconversion risk, assessed via Fine-Gray subdistribution hazards model.

**Results:**

Among 383 participants with sporadic PD, 130 were female, 253 were male, and the mean (SD) age at onset was 61.3 (9.9) years for *MC1R* loss-of-function carriers and 64.6 (10.5) for noncarriers. *MC1R* loss-of-function carriers exhibited 30% faster motor decline (β, 0.57 points/year; 95% CI, 0.16-0.98; *P* = .006) than noncarriers. In the replication cohort, *MC1R* loss-of-function carriers exhibited 50% faster motor decline than noncarriers (β, 1.37; 95% CI, 0.28-2.46; *P* = .01). In a small prodromal cohort, *MC1R* loss-of-function carriers showed a more than 4-fold increased risk of phenoconversion to PD (subdistribution hazard ratio, 4.75; 95% CI, 1.48-15.27; *P* = .009).

**Conclusions and Relevance:**

The findings of this cohort study suggest that *MC1R* loss-of-function variants may define a large, readily identifiable genetic subgroup with accelerated progression, highlighting their utility for potential prognostic stratification and clinical trial enrichment in patients of European descent with PD.

## Key Points

**Question:**

Are common melanocortin 1 receptor (*MC1R*) loss-of-function variants associated with accelerated progression in Parkinson disease (PD)?

**Findings:**

In this cohort study of 383 participants with sporadic PD and 12 years of follow-up, *MC1R* loss-of-function carriers exhibited faster motor decline than noncarriers, and these findings were independently replicated in a pooled cohort of 587 participants from 3 clinical trials. In addition, *MC1R* loss-of-function carriers showed an increased risk of phenoconversion to PD in a prodromal cohort.

**Meaning:**

These findings suggest that *MC1R* loss-of-function variants may define a large and readily genotypable patient subgroup with accelerated motor decline.

## Teaser

This cohort study assesses the association between common *MC1R* variants and Parkinson disease progression.

## Introduction

Heterogeneity in the clinical course of Parkinson disease (PD) remains poorly understood. Pathogenic variants in *LRRK2* and *GBA* influence disease susceptibility and progression, but account for only a fraction of this variability.^1,2,3^ Identifying additional genetic modifiers can improve prognostic accuracy, symptom management, and therapeutic strategies.

Melanocortin 1 receptor (*MC1R*), a key regulator of skin and hair pigmentation through cyclic adenosine monophosphate–dependent signaling, also modulates oxidative stress responses and cytoprotective pathways.^4,5^ Loss-of-function variants in *MC1R* impair eumelanin synthesis and reduce antioxidant capacity.^6^ *MC1R* is expressed in dopaminergic neurons of the substantia nigra, where it may influence neuronal vulnerability.^7^ Previous research^8,9^ showed that *MC1R* disruption compromises the dopaminergic system, while *MC1R* activation is neuroprotective. Peripheral administration of *MC1R* agonists already in clinical use for rare dermatological disorders reduces neuroinflammation and protects against dopaminergic neuron loss.^10,11,12,13^

Phenotypes associated with *MC1R* loss of function, lighter skin, red hair, and melanoma, have each been linked to increased risk of PD, though associations between individual *MC1R* variants and PD risk remain inconsistent.^14,15,16,17,18,19,20,21,22,23,24^ Approximately 60% to 70% of individuals of European descent carry at least 1 *MC1R* loss-of-function variant, with the highest prevalence in Northern European populations.^25^ Despite the high frequency of these variants and converging preclinical and population-based evidence, whether *MC1R* loss-of-function variants influence the course of disease after PD onset remains unknown.

Here, we leveraged longitudinal data from the Parkinson Progression Markers Initiative (PPMI) cohort and a pooled independent replication cohort comprising the Safety of Urate Elevation in Parkinson’s Disease (SURE-PD phase 2),^26^ Study of Urate Elevation in Parkinson’s Disease, Phase 3 (SURE-PD3),^27^ and Efficacy of Isradipine in Early Parkinson Disease III (STEADY-PD III)^28^ cohorts to evaluate whether *MC1R* loss-of-function variants are linked to altered PD progression.

## Methods

### Study Design and Participants

This longitudinal cohort study used data from PPMI, including participants from the PD, genetic registry, and prodromal cohorts.^29^ Participants carrying known pathogenic variants in *SNCA* (n = 39), *PRKN* (n = 9), *PINK1* (n = 1), *PARK7* (n = 1), and *VPS35* (n = 1), as well as those without evidence of dopamine deficiency (n = 66), were excluded. Using genomewide sequencing data generated by the PPMI Genetics Core (Illumina platform processed following Genomic Analysis ToolKit Best Practices), 809 participants with PD were classified as having sporadic (n = 383) or monogenic disease (n = 426) based on *LRRK2* and *GBA* carrier status.^29^ The sporadic cohort served as the primary analysis group. Participants within each group were then stratified by *MC1R* loss-of-function carrier status (sporadic: 231 carriers and 152 noncarriers). Separately, 53 participants with prodromal PD enrolled for rapid eye movement sleep behavior disorder (clinical diagnosis by site investigator, with polysomnography where available) or hyposmia (University of Pennsylvania Smell Identification Test ≤15th age- and sex-adjusted percentile) with available genomewide sequencing data and without *LRRK2* or *GBA* variants were stratified into carriers (n = 34; 21 with rapid eye movement sleep behavior disorder, 13 with hyposmia) and noncarriers (n = 19; 13 with rapid eye movement sleep behavior disorder and 6 with hyposmia).

*MC1R* loss-of-function variants were defined as coding variants with experimentally demonstrated reductions in *MC1R* function.^5,6,16,20^ Variants were further classified by penetrance: high-penetrance R variants substantially reduce *MC1R* function, while low-penetrance r variants reduce function to a lesser extent.^5,6^ *MC1R* loss-of-function carriers were further stratified by zygosity (heterozygosity [including compound heterozygosity] vs homozygosity), variant penetrance, and variant.

Data were collected by PPMI between July 7, 2010, and January 1, 2026, and last accessed via the PPMI portal on February 14, 2026. Informed consent was obtained from all PPMI participants at their respective enrollment sites as part of the PPMI study protocol.

This study followed the Strengthening the Reporting of Observational Studies in Epidemiology (STROBE) reporting guideline. The study was determined to be exempt from institutional review board review at Massachusetts General Hospital as it was a secondary analysis of existing deidentified data.

### Clinical, Imaging, and Biomarker Data

PPMI participants underwent clinical assessments and imaging on recruitment and during subsequent follow-up visits as previously described.^29,30^ Motor decline was assessed using the Movement Disorder Society Unified Parkinson’s Disease Rating Scale Part III (MDS-UPDRS III) over up to 12 years of follow-up (mean [SD] follow-up, 6.8 [3.8] years).^31^ Off- and on-state assessments were analyzed separately. MDS-UPDRS III motor subscores were defined as follows: bradykinesia (items 3.4-3.8, 3.9, 3.14), rigidity (3.3), tremor (3.15-3.18), axial (3.9-3.13), and orofacial (3.1-3.2).^31^ Phenoconversion was defined as a clinician diagnosis of PD, sustained at subsequent visits.

Nonmotor and self-reported motor (MDS-UPDRS Parts I, II, and IV), cognitive (Montreal Cognitive Assessment [MoCA]), autonomic (Scales for Outcomes in Parkinson Disease–Autonomic Dysfunction [SCOPA-AUT]), daytime sleepiness (Epworth Sleepiness Scale [ESS]), and depression (Geriatric Depression Scale [GDS]) outcomes as well as dopamine transporter single-photon emission computed tomography (DAT-SPECT) were assessed as secondary measures of disease progression.^32,33^ Baseline cerebrospinal fluid (CSF) biomarkers, including α-synuclein, tau, phosphorylated tau (p-tau), amyloid β, neurofilament light chain (NfL), and glial fibrillary acidic protein (GFAP), as well as CSF α-synuclein seed amplification assay (SAA) kinetic measures were compared across *MC1R* loss-of-function carriers and noncarriers in the PPMI cohort.^34^

### Statistical Analysis

Linear mixed-effects models with years since baseline as the time scale were used to estimate the yearly rate of change in MDS-UPDRS III, the primary outcome measure, as well as MDS-UPDRS I, II, and IV, MoCA, SCOPA-AUT, ESS, GDS, and DAT-SPECT specific binding ratio (SBR). The primary exposure was *MC1R* loss-of-function carrier status, and each outcome was modeled independently. Group-specific rates of change were estimated using a group-by-time interaction term. Separate models were used for zygosity, penetrance, and individual variant analyses. All models included participant-level random intercepts and slopes for time from baseline visit with an unstructured covariance matrix and allowed for group-specific residual variance. Models were adjusted for age at onset, sex, race (self-reported), baseline assessment score, and concurrent levodopa equivalent daily dose (MDS-UPDRS III models only). Covariate-by-time interactions were evaluated in sensitivity analyses. Estimates were centered at the mean of each covariate except for levodopa equivalent daily dose, which was centered at 0.

Participants were classified as having tremor-dominant or postural instability/gait difficulty motor subtypes at baseline based on the ratio of MDS-UPDRS tremor to postural instability/gait difficulty item scores, and the association between *MC1R* loss-of-function carrier status and motor decline was tested within each subtype.^35^ Enrichment of *MC1R* loss-of-function carriers among fast progressors was assessed using logistic regression with carrier status as the outcome and ordinal progression category as the predictor. Three-way interaction models (*MC1R* × covariate × time) were used to test whether the association between *MC1R* loss-of-function carrier status and motor decline was modified by age at onset, sex, or disease duration. To assess potential population stratification, principal components were computed from genomewide common variants, and the primary analysis was repeated after restriction to genetically defined European participants and adjusted for principal components 1 through 10.

The association between *MC1R* loss-of-function carrier status and rate of change in off-state MDS-UPDRS III score was the primary analysis. Other analyses, including secondary outcomes, biomarkers, motor subscores, and interactions, were exploratory and hypothesis generating. As such, no adjustment for multiple comparisons was applied. A nominal significance level of α = .05 was used for all analyses.

Differences in the distribution of baseline biomarkers were assessed using Wilcoxon rank sum tests. Time to phenoconversion from prodromal to clinically diagnosed PD was analyzed using a Fine-Gray proportional subdistribution hazards model adjusted for baseline age and sex, with phenoconversion to dementia with Lewy bodies or multiple system atrophy treated as a competing event. All analyses were performed in R version 4.4.1 (R Foundation) using nlme version 3.1-164 and survival version 3.6-4.

### Independent Replication Cohort

The replication cohort comprised 587 participants from SURE-PD phase 2^26^ (34 *MC1R* loss-of-function carriers and 40 noncarriers; data collected from June 2009 to December 2012), SURE-PD3^27^ (167 *MC1R* loss-of-function carriers and 59 noncarriers; data collected from June 2016 to June 2019), and STEADY-PD III^28^ (209 *MC1R* loss-of-function carriers and 78 noncarriers; data collected from November 2014 to November 2018) with up to 3 years of follow-up (mean [SD] follow-up, 1.35 [0.92] years). SURE-PD3 and STEADY-PD III participants were genotyped by genomewide sequencing. SURE-PD phase 2 participants were genotyped via the Global Parkinson’s Genetics Program (GP2) Neurobooster Array with BEAGLE imputation against the GP2 release 10 reference panel. V60L and rare variants were below the threshold for reliable imputation. Data were last accessed through the GP2 (gp2.org) platform on March 11, 2026.

The agents used in SURE-PD phase 2,^26^ SURE-PD3,^27^ and STEADY-PD III^28^ did not demonstrate disease modification. SURE-PD phase 2^26^ and STEADY-PD III^28^ administered the UPDRS III, which was converted to MDS-UPDRS III scores using Hoehn & Yahr stage-specific calibration.^26,28,31^ SURE-PD3^27^ administered the MDS-UPDRS III. Visits after initiation of levodopa or dopamine agonist therapy were excluded. Motor decline was modeled using linear mixed-effects models adjusted for age at onset, sex, race (self-reported), baseline score, concurrent monoamine oxidase B inhibitor use, source cohort, and treatment arm assignment.

## Results

### Demographic and Clinical Characteristics of Participants With PD

We analyzed 809 PPMI participants with PD, including 383 with sporadic PD (231 *MC1R* loss-of-function carriers and 152 noncarriers) and 426 with monogenic PD. Demographic and clinical characteristics for the sporadic and monogenic cohorts are displayed in Table 1 and eTable 1 in Supplement 1, respectively. In the sporadic cohort, *MC1R* loss-of-function carriers were younger at disease onset (mean [SD] age, 61.3 [9.9] vs 64.6 [10.5] years; *P* = .002) and had lower MDS-UPDRS III off-state scores at baseline (mean [SD], 19.4 [8.8] vs 23.6 [9.9]; *P* < .001). SAA positivity was high in both groups (96.3% vs 92.9%; *P* = .25), though SAA data were unavailable for a larger proportion of noncarriers. Follow-up duration did not differ between groups (mean [SD], 6.7 [4.0] vs 6.8 [3.7] years; *P* = .69).

**Table 1.** Demographic and Clinical Characteristics for Melanocortin 1 Receptor (*MC1R)* Loss-of-Function Carriers and Noncarriers in the Sporadic Cohort

Characteristic^a^	Noncarriers	*MC1R* loss-of-function carriers
Demographic characteristics		
Participants, No.	152	231
*MC1R* loss-of-function homozygosity, No. (%)	NA	20 (8.7)
Age at PD onset, mean (SD), y	64.6 (10.5)	61.3 (9.9)^b^
Male, No. (%)	96 (63.2)	157 (68.0)
Race, No. (%)^c^		
Asian	1 (0.7)	7 (3.0)
Black	2 (1.3)	3 (1.3)
White	138 (90.8)	210 (90.9)
Unknown	11 (7.2)	11 (4.8)
Clinical characteristics		
Follow-up, mean (SD), y	6.7 (4.0)	6.8 (3.7)
MDS-UPDRS III off-state score at baseline, mean (SD)^d^	23.6 (9.9)	19.4 (8.8)^e^
MoCA score at baseline, mean (SD)^f^	27.3 (2.0)	27.1 (2.6)
Taking PD medication at baseline, No. (%)	0	3 (1.3)
Deep brain stimulation treatment, No. (%)	16 (10.5)	25 (10.8)
α-Synuclein SAA, No. (%)		
Positive	91 (59.9)	208 (90.0)^e^
Negative	7 (4.6)	8 (3.5)
Unknown	54 (35.5)	15 (6.5)
History of melanoma, No. (%)	1 (0.7)	5 (2.2)

Abbreviations: MDS-UPDRS III, Movement Disorder Society Unified Parkinson’s Disease Rating Scale Part III; MoCA, Montreal Cognitive Assessment; PD, Parkinson disease; SAA, seed amplification assay.

^a^ Age at onset (*P* = .002), MDS-UPDRS III at baseline (*P* < .001), and SAA distribution (*P* < .001) differed significantly between groups. The SAA difference was likely driven by differential unavailability (35.5% vs 6.5% unknown); positivity among those tested did not differ (*P* = .25). Sex (*P* = .38), race (*P* = .34), MoCA score (*P* = .80), medication status (*P* = .28), deep brain stimulation treatment (*P* > .99), melanoma history (*P* = .41), and follow-up duration (*P* = .69) did not differ. *P* values compare *MC1R* loss-of-function carriers vs noncarriers in the sporadic cohort. Continuous variables compared using Wilcoxon rank sum test; categorical variables compared using Fisher exact test.

^b^ *P* < .01.

^c^ Race data were collected via participant self-report using fixed categories and reported because *MC1R* variant frequencies differ across ancestral populations, necessitating adjustment for population substructure in progression models.

^d^ MDS-UPDRS Part III motor examination score (range, 0-132; higher scores indicate greater motor impairment).

^e^ *P* < .001.

^f^ Montreal Cognitive Assessment score (range, 0-30; lower scores indicate worse cognitive performance).

Of the 809 participants analyzed, 505 (231/383 [60.3%] sporadic, 187/283 [66.1%] *LRRK2*, 87/143 [60.8%] *GBA*) carried at least 1 *MC1R* loss-of-function variant. *MC1R* loss-of-function carrier frequencies varied across replication cohorts (47–73%), potentially reflecting differences in genotyping methods and population composition. Variant frequencies are summarized in eTables 2 and 3 in Supplement 1.

### *MC1R* Loss-of-Function Carriership and Accelerated Motor Decline

**Figure 1.**
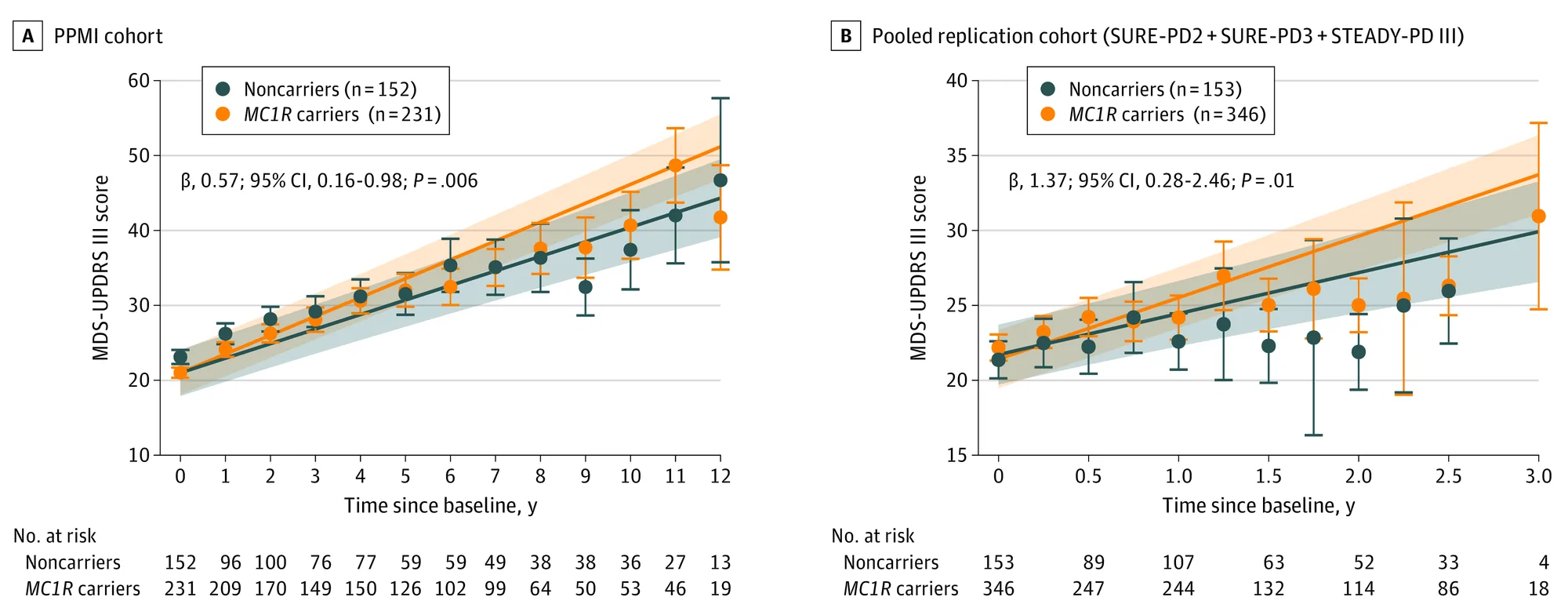
Line Graphs Showing Annual Rate of Motor Decline in Melanocortin 1 Receptor (*MC1R)* Loss-of-Function Carriers vs Noncarriers Change in off-state Movement Disorder Society Unified Parkinson’s Disease Rating Scale Part III (MDS-UPDRS III) score adjusted for age at onset, sex, race, levodopa equivalent daily dose, and baseline score in *MC1R* loss-of-function carriers and noncarriers in the Parkinson Progression Markers Initiative (PPMI) cohort and independent replication cohort (SURE-PD phase 2,^26^ SURE-PD3,^27^ and STEADY-PD III^28^). In the PPMI cohort, the decline was 1.94 points per year among noncarriers and 2.51 points per year among *MC1R* carriers (29.5% faster among *MC1R* carriers). In the pooled replication cohort, the decline was 2.73 points per year among noncarriers and 4.10 points per year among *MC1R* carriers (50.1% faster among *MC1R* carriers). Lines represent the predicted value over time after adjustment. Data points represent the unadjusted mean observed value at the corresponding time point. Shading and error bars represent 95% CIs for observed and predicted values. All statistics are derived from the adjusted model.

In off-state MDS-UPDRS III assessments, *MC1R* loss-of-function carriers exhibited a 30% faster rate of motor decline than noncarriers (2.51 vs 1.94 points/year; β, 0.57; 95% CI, 0.16-0.98; *P* = .006) (Figure 1A; Table 2). Subscore analyses revealed that the accelerated decline was characterized by worsening bradykinesia (1.27 vs 0.94 points/year; β, 0.33; 95% CI, 0.13-0.54; *P* = .001) and orofacial function (0.18 vs 0.14 points/year; β, 0.04; 95% CI, 0.00-0.07; *P* = .03) with no significant differences in tremor, rigidity, or axial subscores. Further, *MC1R* loss-of-function carriers were enriched among fast progressors, with odds ratios (95% CIs) of 1.38 (0.88-2.17) at the median, 1.53 (0.89-2.68) at the top quartile, and 2.09 (0.90-5.49) at the top decile (*P* for trend = .05) (eTable 4 in Supplement 1).

**Table 2.** Annual Rate of Motor Decline in Melanocortin 1 Receptor (*MC1R*) Loss-of-Function Carriers vs Noncarriers in the Sporadic Cohort

Variable^a^	Slope (95% CI)		Difference		*P* value
	Noncarriers (n = 152)	*MC1R* loss-of-function carriers (n = 231)	β (95% CI)	%	
MDS-UPDRS III off-state score^b^	1.94 (1.59 to 2.29)	2.51 (2.25 to 2.78)	0.57 (0.16 to 0.98)	29.5	.006
Tremor	0.24 (0.12 to 0.36)	0.31 (0.21 to 0.40)	0.07 (−0.08 to 0.21)	27.4	.36
Rigidity	0.33 (0.24 to 0.41)	0.35 (0.28 to 0.41)	0.02 (−0.08 to 0.12)	6.4	.68
Bradykinesia	0.94 (0.76 to 1.12)	1.27 (1.14 to 1.41)	0.33 (0.13 to 0.54)	35.5	.001
Axial	0.35 (0.28 to 0.42)	0.36 (0.31 to 0.42)	0.02 (−0.07 to 0.10)	4.8	.70
Orofacial	0.14 (0.11 to 0.17)	0.18 (0.15 to 0.20)	0.04 (0.00 to 0.07)	25.3	.03
MDS-UPDRS III on-state score	0.84 (0.45 to 1.22)	1.09 (0.80 to 1.37)	0.25 (−0.22 to 0.72)	30.0	.30
Tremor	−0.20 (−0.31 to −0.09)	−0.10 (−0.18 to −0.02)	0.10 (−0.04 to 0.23)	48.2	.16
Rigidity	0.11 (0.02 to 0.19)	0.10 (0.04 to 0.16)	−0.01 (−0.11 to 0.10)	−7.9	.88
Bradykinesia	0.44 (0.25 to 0.63)	0.56 (0.43 to 0.69)	0.12 (−0.11 to 0.35)	27.2	.30
Axial	0.40 (0.29 to 0.50)	0.43 (0.35 to 0.50)	0.03 (−0.10 to 0.16)	7.2	.66
Orofacial	0.12 (0.08 to 0.15)	0.12 (0.10 to 0.15)	0.01 (−0.04 to 0.05)	4.5	.80

Abbreviation: MDS-UPDRS III, Movement Disorder Society Unified Parkinson’s Disease Rating Scale Part III.

^a^ Slopes (units per year) were estimated from linear mixed-effects models with time, group, and their interaction as fixed effects, adjusted for age at onset, sex, race, baseline MDS-UPDRS III score, and levodopa equivalent daily dose. Models included participant-level random intercepts and slopes with unstructured covariance and group-specific residual variance.

^b^ MDS-UPDRS III motor subscores were defined as: bradykinesia (items 3.4-3.8, 3.9, 3.14), rigidity (items 3.3a-3.3e), tremor (items 3.15-3.18), axial (items 3.9-3.13), and orofacial (items 3.1-3.2).

The association between *MC1R* loss-of-function carrier status and motor decline was allele dose dependent, with those with homozygosity (n = 20) exhibiting a 63% faster rate of decline (β, 1.16; 95% CI, 0.11-2.22; *P* = .03) compared to 27% in heterozygotes (n = 211; β, 0.53; 95% CI, 0.12-0.95; *P* = .01; *P* for trend = .003) (Table 3). A penetrance gradient was observed across noncarriers, low-penetrance r carriers, and high-penetrance R carriers (1.94, 2.45, and 2.53 points/year, respectively; ordinal trend *P* = .01), with R carriers (n = 111) exhibiting 32% faster decline (β, 0.61; 95% CI, 0.14-1.07; *P* = .01) and r carriers (n = 120) exhibiting 27% faster decline (β, 0.52; 95% CI, 0.03-1.01; *P* = .04). In variant-specific analyses, *R160W* (n = 44; β, 0.70; 95% CI, 0.07-1.34; *P* = .03) and *V92M* (n = 55; β, 0.63; 95% CI, 0.03-1.24; *P* = .04) carriers exhibited significantly faster motor decline. Individuals with all other variants exhibited nominally faster decline (Table 3).

**Table 3.** Annual Rate of Motor Decline (MDS-UPDRS III Off-State) in Melanocortin 1 Receptor (*MC1R*) Loss-of-Function Carriers vs Noncarriers in the Sporadic Cohort Stratified by Zygosity, Penetrance, and Variant

Variable^a^	*MC1R* carrier, No.	*MC1R* loss-of-function carrier, slope (95% CI)	Difference		*P* value
			β (95% CI)	%	
Zygosity					
Heterozygous	211	2.48 (2.21 to 2.75)	0.53 (0.12 to 0.95)	27.3	.01
Homozygous	20	3.02 (2.01 to 4.04)	1.16 (0.11 to 2.22)	62.6	.03
Penetrance^b^					
r Variant	120	2.45 (2.07 to 2.82)	0.52 (0.03 to 1.01)	27.0	.04
R Variant	111	2.53 (2.16 to 2.89)	0.61 (0.14 to 1.07)	31.5	.01
Variant					
R163Q	41	2.27 (1.68 to 2.86)	0.35 (−0.31 to 1.00)	18.0	.30
V60L	78	2.38 (1.94 to 2.82)	0.49 (−0.05 to 1.02)	25.6	.08
V92M	55	2.56 (2.04 to 3.08)	0.63 (0.03 to 1.24)	32.9	.04
R151C	50	2.17 (1.65 to 2.70)	0.35 (−0.25 to 0.94)	18.9	.26
R160W	44	2.63 (2.07 to 3.20)	0.70 (0.07 to 1.34)	36.4	.03
D294N/H	11	2.95 (1.79 to 4.10)	1.07 (−0.12 to 2.25)	56.7	.08

Abbreviation: MDS-UPDRS III, Movement Disorder Society Unified Parkinson’s Disease Rating Scale Part III.

^a^ Slopes (units per year) were estimated from linear mixed-effects models with time, group, and their interaction as fixed effects, adjusted for age at onset, sex, race, baseline MDS-UPDRS III score, and levodopa equivalent daily dose. Models included participant-level random intercepts and slopes with unstructured covariance and group-specific residual variance. Reference group: noncarriers (n = 152; primary model slope = 1.94 points/y).

^b^ R variants are high penetrance while r variants are low penetrance. Pairwise comparisons were not run for D84E (n = 4), R142H (n = 5), A218T (n = 1), R213W (n = 2), R306C (n = 1), C289R (n = 1), and N29KfsTer14 (n = 1) due to sample size limitations.

The association between *MC1R* loss-of-function carrier status and motor decline was consistent across age at onset (β for interaction, −0.002; *P* for interaction = .94), sex (β for interaction, −0.13; *P* for interaction = .77), disease duration (β for interaction, −0.10; *P* for interaction = .80), and tremor-dominant and postural instability/gait difficulty subtypes (eTable 5 in Supplement 1). Further, the association between *MC1R* loss-of-function carrier status and motor decline was stable across the follow-up period, with no evidence of acceleration or attenuation over time (β for interaction, −0.007; quadratic interaction *P* = .79). Results were also robust to inclusion of covariate × time interactions (β, 0.52; 95% CI, 0.11-0.93; *P* = .01). Results were consistent when restricted to genetically European participants (n = 312; β, 0.50; 95% CI, 0.08-0.93; *P* = .02) and when adjusted for principal components (n = 326 β, 0.48; 95% CI, 0.07-0.90; *P* = .02) (eFigure 1 in Supplement 1). Six carriers were subsequently rediagnosed with non-PD conditions compared to 0 noncarriers (6 of 231 [2.6%] vs 0 of 152 [0%]; Fisher exact *P* = .18). Results were consistent after excluding rediagnosed participants (β, 0.51; 95% CI, 0.11-0.91; *P* = .01).

MDS-UPDRS III on-state analyses were consistent with the primary finding but did not reach statistical significance (1.09 vs 0.84 points/year; β, 0.25; 95% CI, −0.22 to 0.72; *P* = .30). In a bivariate model jointly estimating off- and on-state trajectories, medication substantially attenuated observed motor decline (state × time β, −0.84; 95% CI, −1.04 to −0.64; *P* < .001), with off-state slopes nearly 2-fold steeper than on-state slopes (1.96 vs 1.11 points/year in noncarriers; 2.59 vs 1.48 in carriers).

When all participants were pooled across the sporadic and monogenic cohorts and adjusted for *LRRK2* and *GBA* status, *MC1R* loss-of-function carriers (n = 505) exhibited 25% faster motor decline than noncarriers (n = 304; β, 0.48; 95% CI, 0.13-0.83; *P* = .008), with the association again characterized by bradykinesia (1.18 vs 0.93 points/year; β, 0.25; 95% CI, 0.07-0.42; *P* = .006) and orofacial function (0.17 vs 0.12 points/year; β, 0.05; 95% CI, 0.02-0.08; *P* = .001) (eTable 6 in Supplement 1). The association between *MC1R* loss-of-function carrier status and motor decline did not differ by *GBA* (β for interaction, −0.59; *P* for interaction = .26) or *LRRK2* (β for interaction, 0.03; *P* for interaction = .95) status, and in stratified analyses was nominally consistent in *LRRK2* variant carriers (2.13 vs 1.64 points/year; β, 0.49; 95% CI, −0.25 to 1.22; *P* = .20) and null in *GBA* variant carriers (2.46 vs 2.56 points/year; β, −0.09; 95% CI, −1.29 to 1.10; *P* = .88) (eTable 7 in Supplement 1).

No significant differences between *MC1R* loss-of-function carriers and noncarriers were observed for MDS-UPDRS Parts I, II, or IV, MoCA, SCOPA-AUT, GDS, ESS, or DAT-SPECT SBR (eTable 8 in Supplement 1). Annual rates of change in MoCA and DAT-SPECT SBR were modest across both groups (MoCA: −0.12 vs −0.20 points/year; putamen SBR: −0.07 vs −0.07/year in noncarriers vs carriers), limiting our ability to detect between-group differences. Baseline CSF biomarkers including α-synuclein, tau, p-tau, amyloid β, NfL, GFAP, and α-synuclein SAA kinetic measures did not differ between *MC1R* loss-of-function carriers and noncarriers (eTable 9 in Supplement 1).

### Replication in Independent Clinical Trial Cohorts

Demographic and clinical characteristics for the replication cohort are displayed in eTable 10 in Supplement 1. In the replication cohort, *MC1R* loss-of-function carriers (n = 346 excluding 64 *LRRK2/GBA* variant carriers) exhibited a 50% faster rate of motor decline than noncarriers (n = 153 excluding 24 *LRRK2/GBA* carriers; 4.10 vs 2.73 points/year; β, 1.37; 95% CI, 0.28-2.46; *P* = .01), consistent with our findings in PPMI (Figure 1B). When *LRRK2/GBA* carriers were included and adjusted for, the association remained significant (n = 410 *MC1R* loss-of-function carriers and 177 noncarriers; β, 1.23; 95% CI, 0.22-2.24; *P* = .02). The distribution of individual progression rates and their relationship to time to treatment initiation in the PPMI and the replication cohorts are shown in eFigures 2 and 3 in Supplement 1.

### *MC1R* Loss-of-Function Carriership and Increased Risk of Phenoconversion

**Figure 2.**
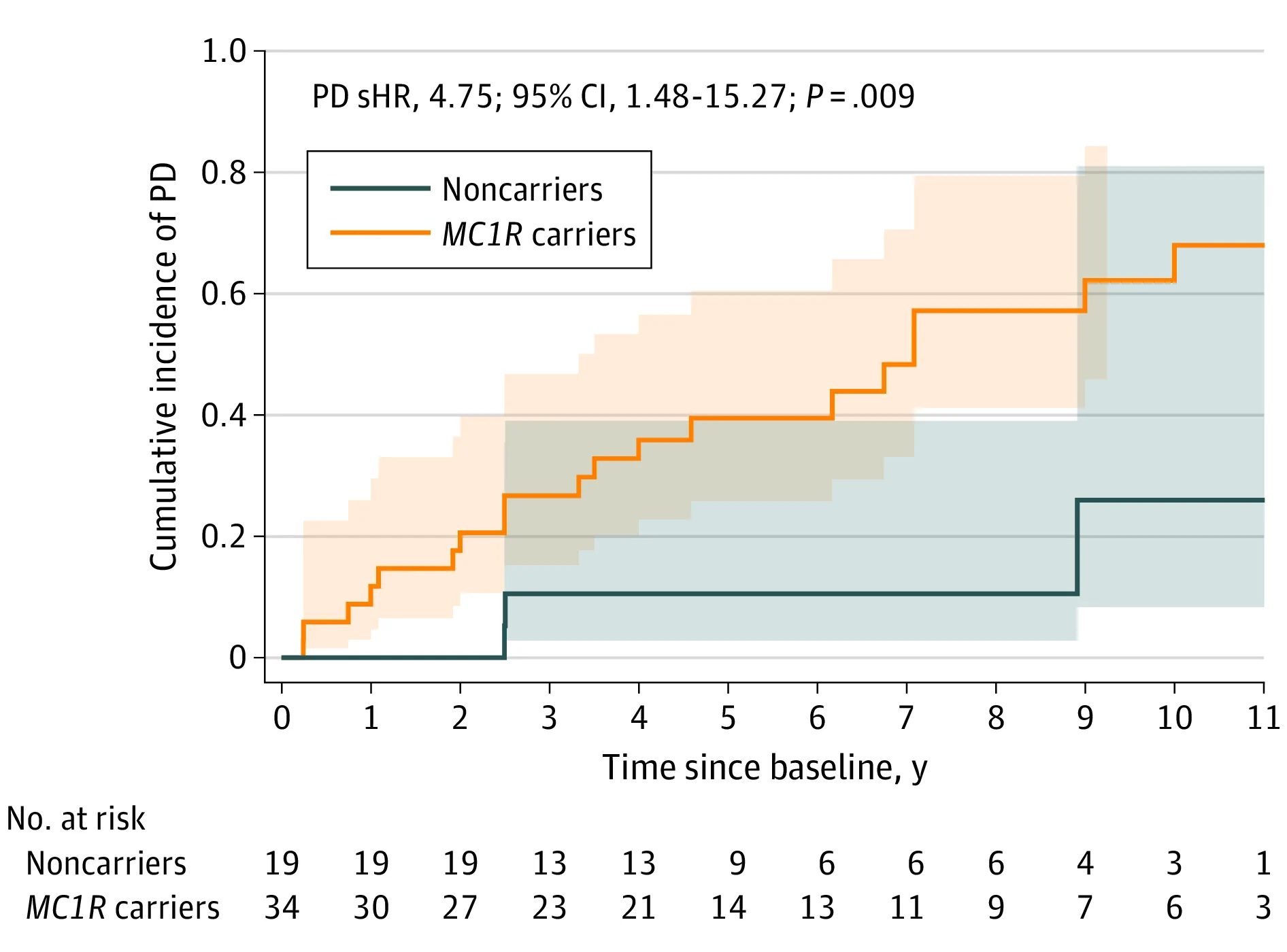
Cumulative Incidence Curve Showing Phenoconversion to Parkinson Disease (PD) in Melanocortin 1 Receptor (*MC1R*) Loss-of-Function Carriers vs Noncarriers The cumulative incidence of phenoconversion to PD in the Parkinson Progression Markers Initiative (PPMI) cohort for noncarriers and *MC1R* loss-of-function carriers with prodromal PD was estimated using a Fine-Gray proportional subdistribution hazards model adjusted for age and sex, with dementia with Lewy bodies or multiple system atrophy phenoconversion as a competing event. sHR indicates subdistribution hazard ratio.

Among the 53 prodromal PPMI participants with available genomewide sequencing data (34 *MC1R* loss-of-function carriers and 19 noncarriers), 23 phenoconverted to clinically diagnosed PD during follow-up (20 *MC1R* loss-of-function carriers and 3 noncarriers) (eTable 11 in Supplement 1). *MC1R* loss-of-function carriers exhibited more than 4-fold increased risk of phenoconversion (PD subdistribution hazard ratio, 4.75; 95% CI, 1.48-15.27; *P* = .009) (Figure 2).

## Discussion

Previous studies examining *MC1R* loss-of-function variants and PD risk have yielded mixed results, with inconsistent associations reported for R160W and R151C.^19,20,21,22,23,24^ In this cohort study of 383 participants with sporadic PD from the PPMI cohort, *MC1R* loss-of-function carriers exhibited a 30% faster rate of motor decline than noncarriers. Findings were replicated in an independent pooled cohort. In addition, prodromal *MC1R* loss-of-function carriers exhibited a more than 4-fold increased risk of phenoconversion to clinically diagnosed PD. *MC1R* loss-of-function variants were present in more than 60% of the participants analyzed in this study.^19,20,21,22,23,24^ Together, these findings identify *MC1R* loss-of-function variants as common genetic modifiers of motor decline in PD.

Accelerated motor decline in *MC1R* loss-of-function carriers appeared to be confined to bradykinesia, the motor feature most closely linked to nigrostriatal dopamine loss, and orofacial function, with no significant differences in tremor, rigidity, or axial subscores.^36^ Reduced *MC1R* function shifts systemic melanin synthesis toward pheomelanin, increasing oxidative stress and depleting glutathione, an antioxidant implicated in PD pathogenesis.^6,37,38^ A previous study ^11^ showed that the pheomelanin component of neuromelanin was increased in the substantia nigra of patients with PD, suggesting a link between altered local melanin metabolism and dopaminergic vulnerability. Reduced *MC1R* function also impairs immune regulation and cellular stress response while promoting neuroinflammation.^10,38,39^ The convergence of melanin dysregulation, oxidative damage, and impaired immune responses provides a multifaceted biological framework consistent with enhanced dopaminergic vulnerability and the accelerated motor decline observed in this study.

The observed allele dose-response between *MC1R* loss of function and motor decline, with individuals with homozygosity exhibiting more than twice the effect size of those with heterozygosity, represents a biological gradient difficult to attribute to unmeasured confounders and instead suggests an association between *MC1R* signaling capacity and disease progression. We also observed modest differences based on variant penetrance, with high-penetrance R variants producing larger effect sizes than low-penetrance r variants, consistent with known functional differences in *MC1R* signaling capacity as a result of these variants.^5^ *MC1R* has an established role in the dopa-melanin biosynthetic pathway that may be subject to pharmacological confounding; however, within the PPMI cohort, the association between *MC1R* loss of function and motor decline was strongest in the off-state, where medication influence is minimized, and remained consistent after adjustment for concurrent levodopa equivalent daily dose. Importantly, replication in an independent pooled cohort of patients with de novo PD precluded medication confounding.

### Limitations

This study has limitations. First, the cohorts analyzed here are predominantly of European ancestry.^40^ *MC1R* loss-of-function variants vary across populations, and our findings may not generalize to populations of non-European ancestry or within genetically distinct European subpopulations. Second, the absence of an association between *MC1R* loss-of-function variants, nonmotor outcomes, and imaging may reflect limited sensitivity within the follow-up period rather than true motor specificity, particularly given that MoCA and DAT-SPECT SBR exhibited limited change across groups. Whether *MC1R* influences the rate of nigrostriatal degeneration or instead modulates the clinical expression of motor progression through compensatory or resilience-related mechanisms remains an open question that longer follow-up periods may help resolve. Third, replication cohorts with extended off-state follow-up are rare, a limitation of this study and of the field more broadly. Fourth, the phenoconversion and subgroup analyses, while significant, were limited by sample size. In particular, replication in larger prodromal cohorts ascertained independently of monogenic risk factors is needed. Fifth, PPMI does not collect skin pigmentation or hair color data, and CSF biomarkers at baseline did not differ between groups, limiting insights into relevant pigmentary and nonpigmentary mechanisms. Sixth, neuropathological data were available for 17 participants in the sporadic cohort (9 carriers, 8 noncarriers); however, sample sizes remain too small for meaningful comparison between groups.

## Conclusions

The high prevalence of *MC1R* loss-of-function variants in European populations and the magnitude of their association with motor decline define a large, readily identifiable patient subgroup with accelerated motor decline. Given that *MC1R* carrier status can be determined through simple genotyping, these findings have implications for prognostic stratification, clinical counseling, and enrichment strategies in trial design. The association between *MC1R* loss-of-function variants and disease trajectory observed across patients with prodromal PD, early de novo PD, and those requiring dopaminergic therapy supports a role for *MC1R* in early and ongoing disease biology despite its inconsistent association with disease risk. The findings presented here along with independent preclinical evidence that existing clinical-stage *MC1R* agonists are neuroprotective in models of PD provide a foundation for future investigation of *MC1R* agonists as potential disease-modifying therapies in PD.

## Notes

Supplement 1. eTable 1. Demographics and clinical characteristics for LRRK2, LRRK2 + MC1R, GBA, and GBA + MC1R carriers in the monogenic cohort eTable 2. MC1R LoF variant carrier frequency in the PPMI cohort and independent replication cohorts eTable 3. MC1R LoF variant minor allele frequency in other PD cohorts and gnomAD eTable 4. Enrichment among fast progressors (MDS-UPDRS III OFF-state) in MC1R LoF carriers versus non-carriers in the sporadic cohort eTable 5. Annual rate of motor decline (MDS-UPDRS III OFF-state) in MC1R LoF carriers versus non-carriers in the sporadic cohort stratified by motor subtype eTable 6. Annual rate of motor decline (MDS-UPDRS III OFF-state) in MC1R LoF carriers versus non-carriers in the sporadic and monogenic cohorts combined eTable 7. Annual rate of motor decline (MDS-UPDRS III OFF-state) in LRRK2 carriers versus LRRK2 + MC1R carriers and GBA carriers versus GBA + MC1R carriers eTable 8. Annual progression of non-motor and functional outcomes in MC1R LoF carriers versus non-carriers in the sporadic cohort eTable 9. Baseline CSF biomarker concentrations and SAA kinetic measures in MC1R LoF carriers versus non-carriers in the sporadic cohort eTable 10. SURE-PD phase 2, SURE-PD3, and STEADY-PD III demographics, clinical, and genetic characteristics eTable 11. Demographics for prodromal participants with and without MC1R variants eFigure 1. Principal component analysis of PPMI participants eFigure 2. Distribution of individual motor progression rates and relationship to treatment initiation in the PPMI cohort eFigure 3. Distribution of individual motor progression rates and relationship to treatment initiation in the replication cohort

Supplement 2. Data sharing statement
