# Supplementary material for: Common MC1R Variants and Parkinson Disease Progression
Source: JAMA Neurol. 2026 Sep 8;83(10):981–9. doi: 10.1001/jamaneurol.2026.2998 (PMC13555505; doi:10.1001/jamaneurol.2026.2998)
Supplement: Supplementary file 1 [file jamaneurol-e262998-s001.pdf]

Supplemental Online Content

Schumacher JG, Zhang X, Wang J, et al. Common *MC1R* variants and parkinson disease progression. *JAMA Neurology*. Published online September 9. 2026.
doi:10.1001/jamaneurol.2026.2998

eTable 1. Demographics and clinical characteristics for LRRK2, LRRK2 + MC1R, GBA, and GBA + MC1R carriers in the monogenic cohort
eTable 2. MC1R LoF variant carrier frequency in the PPMI cohort and independent replication cohorts
eTable 3. MC1R LoF variant minor allele frequency in other PD cohorts and gnomAD
eTable 4. Enrichment among fast progressors (MDS-UPDRS III OFF-state) in MC1R LoF carriers versus non-carriers in the sporadic cohort
eTable 5. Annual rate of motor decline (MDS-UPDRS III OFF-state) in MC1R LoF carriers versus non-carriers in the sporadic cohort stratified by motor subtype
eTable 6. Annual rate of motor decline (MDS-UPDRS III OFF-state) in MC1R LoF carriers versus non-carriers in the sporadic and monogenic cohorts combined
eTable 7. Annual rate of motor decline (MDS-UPDRS III OFF-state) in LRRK2 carriers versus LRRK2 + MC1R carriers and GBA carriers versus GBA + MC1R carriers
eTable 8. Annual progression of non-motor and functional outcomes in MC1R LoF carriers versus non-carriers in the sporadic cohort
eTable 9. Baseline CSF biomarker concentrations and SAA kinetic measures in MC1R LoF carriers versus non-carriers in the sporadic cohort
eTable 10. SURE-PD phase 2, SURE-PD3, and STEADY-PD III demographics, clinical, and genetic characteristics
eTable 11. Demographics for prodromal participants with and without MC1R variants
eFigure 1. Principal component analysis of PPMI participants
eFigure 2. Distribution of individual motor progression rates and relationship to treatment initiation in the PPMI cohort
eFigure 3. Distribution of individual motor progression rates and relationship to treatment initiation in the replication cohort

This supplemental material has been provided by the authors to give readers additional information about their work.

© 2026 Schumacher JG et al. *JAMA Neurology*.

eTable 1: Demographics and clinical characteristics for *LRRK2*, *LRRK2 + MC1R*, *GBA*, and *GBA + MC1R* carriers in the monogenic cohort

	***LRRK2* carriers**	***LRRK2 + MC1R* carriers**	***GBA* carriers**	***GBA + MC1R* carriers**
**Demographics**				
Participants	96	187	56	87
MC1R LoF homozygotes (%)	0	16 (8.6)	0	5 (5.7)
Age at PD onset (SD)	62.6 (10.4)	65.1 (10.9)	60.2 (10.3)	61.6 (10.2)
Male (%)	53 (55.2)	87 (46.5)	28 (50.0)	53 (60.9)
Race (%)				
Asian	1 (1.1)	0	0	0
White	80 (83.3)	175 (93.6)	52 (92.9)	85 (97.7)
Unknown	15 (15.6)	12 (6.4)	4 (7.1)	2 (2.3)
**Clinical characteristics**				
Average follow-up (SD)	4.4 (3.2)	3.9 (3.6)	4.0 (3.7)	4.0 (3.3)
MDS-UPDRS III score at baseline (SD)	19.0 (10.6)	21.4 (10.8)	26.2 (14.6)	24.9 (12.1)
MoCA score at baseline (SD)	25.7 (3.1)	26.1 (3.0)	26.1 (3.5)	26.5 (2.6)
On PD medication at baseline (%)	57 (67.1)	97 (66.0)	27 (51.9)	39 (51.3)
α-Synuclein SAA status (%)				
Positive	46 (47.9)	70 (37.4)	29 (51.8)	48 (55.2)
Negative	14 (14.6)	39 (20.9)	0	4 (4.6)
Unknown	36 (37.5)	78 (41.7)	27 (48.2)	35 (40.2)
History of melanoma (%)	0	1 (0.5)	0	1 (1.1)

Statistical analysis comparing group characteristics was not performed. Abbreviations: PD, Parkinson disease; LRRK2, leucine-rich repeat kinase 2; GBA, glucocerebrosidase; MC1R, melanocortin 1 receptor; SD, standard deviation; SAA, seed amplification assay; MDS-UPDRS III, Movement Disorder Society Unified Parkinson's Disease Rating Scale Part III; MoCA, Montreal Cognitive Assessment

© 2026 Schumacher JG et al. *JAMA Neurology*.

eTable 2. *MC1R* LoF variant carrier frequency in the PPMI cohort and independent replication cohorts

	Penetrance	All PPMI (N=809)	PPMI Sporadic Cohort (N=383)	SURE-PD phase 2 (N=74)	SURE-PD3 (N=259)	STEADY-PD III (N=329)
All variants	----	505 (62.4)	231 (60.3)	34 (45.9)	179 (69.1)	234 (71.1)
r variants	----	365 (45.1)	152 (39.7)	17 (23.0)	122 (47.1)	169 (51.4)
R variants	----	199 (24.6)	103 (26.9)	20 (27.0)	83 (32.0)	107 (32.5)
R163Q (rs885479)	r	76 (9.4)	41 (10.7)	4 (5.4)	25 (9.7)	39 (11.9)
V60L (rs1805005)	r	241 (29.8)	78 (20.4)	----	68 (26.3)	90 (27.4)
V92M (rs2228479)	r	95 (11.7)	55 (14.4)	7 (9.5)	35 (13.5)	54 (16.4)
R151C (rs1805007)	R	91 (11.2)	50 (13.1)	6 (8.1)	44 (17.0)	35 (10.6)
R160W (rs1805008)	R	82 (10.1)	44 (11.5)	8 (10.8)	34 (13.1)	49 (14.9)
D294N/H (rs1805009)	R	14 (1.7)	11 (2.9)	1 (1.4)	9 (3.5)	13 (4.0)
D84E (rs1805006)	R	9 (1.1)	4 (1.0)	3 (4.1)	5 (1.9)	7 (2.1)
R142H (rs11547464)	R	17 (2.10)	5 (1.3)	2 (2.7)	2 (0.8)	10 (3.0)
A218T (rs200965363)	R	1 (0.1)	1 (0.3)	----	0	1 (0.3)
R213W (rs200000734)	R	3 (0.4)	2 (0.6)	----	0	0
R306C (rs747847234)	R	1 (0.1)	1 (0.3)	----	0	0
C289R (rs369542041)	R	1 (0.1)	1 (0.3)	----	0	0
N29KfsTer14	R	3 (0.4)	1 (0.3)	----	3 (1.2)	1 (0.3)

Values are N (%). Carrier frequency = proportion of participants carrying ≥1 copy of the variant. R variants = high-penetrance variants and r variants = low-penetrance variants. PPMI, SURE-PD3, and STEADY-PD III genotyped by whole genome sequencing. SURE-PD2 genotyped via GP2 Neurobooster Array with BEAGLE imputation against GP2 release 10 reference panel. V60L was not recovered by imputation due to poor linkage disequilibrium scaffolding on the array. Additional rare variants (A218T, R213W, R306C, C289R, N29KfsTer14) were below the threshold for reliable imputation. Per-variant counts derived from imputed dosages (carrier threshold > 0.5). SURE-PD phase 2, SURE-PD3, and STEADY-PD III frequencies presented before excluding *LRRK2* and *GBA* carriers. Abbreviations: PD, Parkinson disease; MC1R, melanocortin 1 receptor

© 2026 Schumacher JG et al. *JAMA Neurology*.

eTable 3. *MC1R* LoF variant minor allele frequency in other PD cohorts and gnomAD

	Penetrance	Lorenzo-Betancor et al. 2016 (N=889)	Gan-Or et al. 2016 (SPOT) (N=539)	Gan-Or et al. 2016 (MNI) (N=551)	gnomAD EUR (Finnish)	gnomAD EUR (Non-Finnish)	gnomAD AJ	gnomAD EAS	gnomAD AFR
All variants	----	0.47	----	----	----	----	----	----	----
r variants	----	0.27	0.33	0.23	----	----	----	----	----
R variants	----	0.20	----	----	----	----	----	----	----
R163Q (rs885479)	r	0.05	0.06	0.02	0.22	0.05	0.03	0.71	0.02
V60L (rs1805005)	r	0.13	0.20	0.14	0.06	0.12	0.29	<0.01	0.02
V92M (rs2228479)	r	0.09	0.07	0.07	0.07	0.09	0.06	0.19	0.01
R151C (rs1805007)	R	0.09	0.07	0.05	0.07	0.09	0.07	<0.01	0.01
R160W (rs1805008)	R	0.08	0.05	0.05	0.07	0.08	0.06	<0.01	0.01
D294N/H (rs1805009)	R	0.02	0.01	0.02	<0.01	0.02	<0.01	<0.01	<0.01
D84E (rs1805006)	R	0.01	----	----	<0.01	0.01	<0.01	<0.01	<0.01
R142H (rs11547464)	R	0.01	----	----	<0.01	0.01	0.02	<0.01	<0.01
A218T (rs200965363)	R	----	----	----	<0.01	<0.01	<0.01	<0.01	<0.01
R213W (rs200000734)	R	----	----	----	<0.01	<0.01	<0.01	<0.01	<0.01
R306C (rs747847234)	R	----	----	----	<0.01	<0.01	<0.01	<0.01	<0.01
C289R (rs369542041)	R	----	----	----	<0.01	<0.01	<0.01	<0.01	<0.01
N29KfsTer14	R	----	----	----	----	----	----	----	----

All data retrieved from the associated publications and gnomAD. Abbreviations: PD, Parkinson disease; MC1R, melanocortin 1 receptor

© 2026 Schumacher JG et al. *JAMA Neurology*.

eTable 4: Enrichment among fast progressors (MDS-UPDRS III OFF-state) in *MC1R* LoF carriers versus non-carriers in the sporadic cohort

Threshold	N_{SPD} (%)	N_{MC1R} (%)	OR (95% CI)	Fisher p	p-trend
Median	58 (38.2)	122 (53.0)	1.38 (0.88, 2.17)	0.154	
Top quartile	26 (17.1)	64 (27.8)	1.53 (0.89, 2.68)	0.128	0.05
Top decile	8 (5.3)	28 (12.2)	2.09 (0.90, 5.49)	0.098	

Odds ratios for *MC1R* LoF carrier status among patients exceeding MDS-UPDRS III progression thresholds. Individual slopes were derived from linear mixed-effects models with time as a fixed and random effect, adjusted for age at onset, sex, race, baseline MDS-UPDRS III score, and LEDD. Models included participant-level random intercepts and slopes with unstructured covariance. P-trend from logistic regression with ordinal progression category as predictor. Abbreviations: PD, Parkinson disease; MC1R, melanocortin 1 receptor; MDS-UPDRS III, Movement Disorder Society Unified Parkinson's Disease Rating

© 2026 Schumacher JG et al. *JAMA Neurology*.

eTable 5: Annual rate of motor decline (MDS-UPDRS III OFF-state) in *MC1R* LoF carriers versus non-carriers in the sporadic cohort stratified by motor subtype

	$N_{Non\text{-}carrier}$	N_{MC1R}	Non-carrier PD Slope (95% CI)	*MC1R* LoF carrier Slope (95% CI)	β diff (95% CI)	% diff	p-value
Tremor-dominant	70	167	2.09 (1.69, 2.49)	2.49 (2.20, 2.77)	0.40 (-0.06, 0.86)	19.0%	0.09
PIGD	19	34	1.69 (0.85, 2.54)	2.42 (1.69, 3.16)	0.73 (-0.33, 1.78)	43.0%	0.18

93 participants classified as indeterminate motor subtype were excluded from this analysis. Slopes (units per year) were estimated from linear mixed effects models with time, group, and their interaction as fixed effects, adjusted for age at onset, sex, race, baseline MDS-UPDRS III score, and LEDD. Models included participant-level random intercepts and slopes with unstructured covariance and group-specific residual variance. Visit-to-visit subtype volatility was comparable between groups (mean switch rate 0.11 vs 0.13 per visit interval), with 73% of non-carriers and 70% of carriers maintaining a stable classification throughout follow-up. TD-to-PIGD conversion occurred at nearly double the rate in carriers vs non-carriers (23/167 [13.8%] vs 5/71 [7.0%]), though this difference did not reach significance (p=0.19). When restricted to sustained conversions (PIGD at both of the final two assessments), rates were 8.1% in carriers and 4.5% in non-carriers (p=0.41). Abbreviations: PD, Parkinson disease; MC1R, melanocortin 1 receptor; MDS-UPDRS III, Movement Disorder Society Unified Parkinson's Disease Rating Scale Part III; 95% CI, 95% confidence interval

© 2026 Schumacher JG et al. *JAMA Neurology*.

eTable 6. Annual rate of motor decline (MDS-UPDRS III OFF-state) in *MC1R* LoF carriers versus non-carriers in the sporadic and monogenic cohorts combined

	Non-carrier Slope (95% CI)	*MC1R* LoF carrier Slope (95% CI)	β diff (95% CI)
**MDS-UPDRS III OFF**	1.91 (1.63, 2.20)	2.39 (2.17, 2.61)	0.48 (0.13, 0.83
Tremor	0.25 (0.15, 0.34)	0.29 (0.21, 0.36)	0.04 (-0.08, 0.16
Rigidity	0.32 (0.26, 0.39)	0.36 (0.31, 0.41)	0.04 (-0.04, 0.12
Bradykinesia	0.93 (0.78, 1.07)	1.18 (1.06, 1.29)	0.25 (0.07, 0.42
Axial	0.33 (0.27, 0.39)	0.37 (0.33, 0.42)	0.05 (-0.03, 0.12
Orofacial	0.12 (0.04, 0.14)	0.17 (0.15, 0.19)	0.05 (0.02, 0.08
**MDS-UPDRS III ON**	0.77 (0.50, 1.04)	0.99 (0.78, 1.19)	0.21 (-0.12, 0.55
Tremor	-0.12 (-0.20, -0.05)	-0.11 (-0.17, -0.06)	0.01 (-0.08, 0.10
Rigidity	0.11 (0.05, 0.17)	0.10 (0.05, 0.14)	-0.01 (-0.09, 0.07
Bradykinesia	0.39 (0.25, 0.52)	0.53 (0.43, 0.63)	0.14 (-0.02, 0.31
Axial	0.36 (0.28, 0.43)	0.43 (0.37, 0.48)	0.07 (-0.02, 0.16
Orofacial	0.11 (0.08, 0.13)	0.13 (0.11, 0.14)	0.02 (-0.01, 0.05

Slopes (units per year) were estimated from linear mixed effects models with time, group, and their interaction as fixed effects, adjusted for age at onset, sex, race, *LRRK2/GBA* status, baseline MDS-UPDRS III score, and LEDD. Models included participant-level random intercepts and slopes with unstructured covariance and group-specific residual variance. n=304 non-carriers and 505 *MC1R* LoF carriers. MDS-UPDRS III motor subscores were defined as: bradykinesia (items 3.4-3.8, 3.9, 3.14), rigidity (items 3.3a-3.3e), tremor (items 3.15-3.18), axial (items 3.9-3.13), and orofacial (items 3.1-3.2). Abbreviations: PD, Parkinson disease; LRRK2, leucine-rich repeat kinase 2; GBA, glucocerebrosidase; MC1R, melanocortin 1 receptor; MDS-UPDRS III, Movement Disorder Society Unified Parkinson's Disease Rating Scale Part III; 95% CI, 95% confidence interval

eTable 7. Annual rate of motor decline (MDS-UPDRS III OFF-state) in *LRRK2* carriers versus *LRRK2* + *MC1R* carriers and *GBA* carriers versus *GBA* + *MC1R* carriers

	***LRRK2* Slope (95% CI)**	***LRRK2* + *MC1R* Slope (95% CI)**	**β diff (95% CI**
**MDS-UPDRS III OFF**	1.64 (1.06, 2.22)	2.13 (1.65, 2.61)	0.49 (-0.25, 1.2
Tremor	0.19 (-0.01, 0.38)	0.26 (0.10, 0.42)	0.08 (-0.17, 0.3
Rigidity	0.31 (0.19, 0.43)	0.38 (0.28, 0.49)	0.07 (-0.08, 0.2
Bradykinesia	0.74 (0.43, 1.05)	0.98 (0.73, 1.24)	0.25 (-0.15, 0.6
Axial	0.29 (0.17, 0.41)	0.33 (0.23, 0.43)	0.04 (-0.11, 0.1
Orofacial	0.08 (0.04, 0.13)	0.14 (0.10, 0.18)	0.06 (0.00, 0.1
	***GBA* Slope (95% CI)**	***GBA* + *MC1R* Slope (95% CI)**	
**MDS-UPDRS III OFF**	2.56 (1.61, 3.50)	2.46 (1.70, 3.23)	-0.09 (-1.29, 1.1
Tremor	0.36 (0.03, 0.70)	0.24 (-0.04, 0.51)	-0.13 (-0.56, 0.3
Rigidity	0.30 (0.07, 0.52)	0.34 (0.16, 0.52)	0.04 (-0.24, 0.3
Bradykinesia	1.34 (0.89, 1.79)	1.18 (0.81, 1.55)	-0.17 (-0.73, 0.3
Axial	0.35 (0.16, 0.54)	0.53 (0.38, 0.69)	0.19 (-0.06, 0.4
Orofacial	0.11 (0.02, 0.20)	0.20 (0.13, 0.27)	0.09 (-0.02, 0.2

Slopes (units per year) were estimated from linear mixed effects models with time, group, and their interaction as fixed effects, adjusted for age at onset, sex, race, baseline MDS-UPDRS III score, and LEDD. Models included participant-level random intercepts and slopes with unstructured covariance and group-specific residual variance. n=96 *LRRK2* carriers, 187 *LRRK2 + MC1R* carriers, 56 *GBA* carriers, and 87 *GBA + MC1R* carriers. MDS-UPDRS III motor subscores were defined as: bradykinesia (items 3.4-3.8, 3.9, 3.14), rigidity (items 3.3a-3.3e), tremor (items 3.15-3.18), axial (items 3.9-3.13), and orofacial (items 3.1-3.2). Abbreviations: PD, Parkinson disease; LRRK2, leucine-rich repeat kinase 2; GBA, glucocerebrosidase; MC1R, melanocortin 1 receptor; MDS-UPDRS III, Movement Disorder Society Unified Parkinson's Disease Rating Scale Part III; 95% CI, 95% confidence interval

eTable 8. Annual progression of non-motor and functional outcomes in *MC1R* LoF carriers versus non-carriers in the sporadic cohort

	Non-carrier Slope (95% CI)	*MC1R* carrier Slope (95% CI)	β diff (95% CI)
**Longitudinal outcomes**			
MDS-UPDRS I	0.42 (0.33, 0.51)	0.50 (0.44, 0.56)	0.08 (-0.03, 0.19
MDS-UPDRS II	0.93 (0.72, 1.14)	1.06 (0.91, 1.20)	0.12 (-0.14, 0.38
MDS-UPDRS IV	0.39 (0.29, 0.49)	0.39 (0.32, 0.46)	0.00 (-0.12, 0.12
MoCA	-0.22 (-0.32, -0.12)	-0.20 (-0.27, -0.13)	0.02 (-0.10, 0.14
SCOPA-AUT	0.70 (0.58, 0.83)	0.66 (0.57, 0.75)	-0.04 (-0.19, 0.11
GDS	0.10 (0.03, 0.16)	0.15 (0.11, 0.20)	0.06 (-0.02, 0.14
ESS	0.26 (0.17, 0.35)	0.33 (0.26, 0.39)	0.07 (-0.05, 0.18
**Dopaminergic imaging**			
DAT-SPECT Caudate SBR	-0.13 (-0.15, -0.11)	-0.13 (-0.15, -0.12)	0.00 (-0.02, 0.03
DAT-SPECT Putamen SBR	-0.07 (-0.08, -0.06)	-0.07 (-0.08, -0.06)	0.00 (-0.01, 0.02

Slopes (units per year) were estimated from linear mixed effects models with time, group, and their interaction as fixed effects, adjusted for age at onset, sex, race, and baseline outcome score. Models included participant-level random intercepts and slopes with unstructured covariance and group-specific residual variance. n=231 *MC1R* LoF carriers and 152 non-carriers for longitudinal outcomes; 220 *MC1R* carriers and 100 non-carriers for dopaminergic imaging. Abbreviations: PD, Parkinson disease; MC1R, melanocortin 1 receptor; 95% CI, 95% confidence interval; MDS-UPDRS I, II, and IV, Movement Disorder Society Unified Parkinson's Disease Rating Scale Part I, II, and IV; MoCA, Montreal Cognitive Assessment; SCOPA-AUT, Scales for Outcomes in Parkinson's disease - Autonomic Dysfunction; GDS, Geriatric Depression Scale; ESS, Epworth Sleepiness Scale; DAT-SPECT, dopamine transporter imaging with single-photon emission computed tomography; SBR, specific binding ratio

eTable 9. Baseline CSF biomarker concentrations and SAA kinetic measures in *MC1R* LoF carriers versus non-carriers in the sporadic cohort

Analyte	N_{SPD}	N_{MC1R}	Non-carrier mean (SD)	*MC1R* LoF carrier mean (SD)
**CSF Biomarkers (pg/ml)**				
Alpha synuclein	278	518	106.5 (54.9)	107.9 (49.8)
Tau	97	207	169.5 (60.6)	171.2 (56.2)
p-Tau	90	194	15.0 (5.4)	15.1 (5.3)
Amyloid beta	96	215	971.1 (434.2)	893.8 (384.7)
NfL	394	692	23.6 (40.4)	23.0 (40.8)
GFAP	277	494	39.9 (50.7)	42.5 (54.7)
**CSF SAA kinetic measures**				
Time to threshold (hours)	104	189	66.7 (15.2)	66.2 (12.0)
Time to 50% threshold (hours)	104	189	72.0 (13.6)	71.4 (11.4)
Fluorescence maximum (RFU)	104	189	74179 (34271)	78219 (29631)
Area under the curve (RFU x hours)	104	189	2.6×10^7 (4.8×10^6)	2.6×10^7 (4.1×10^6)

Reported values are mean (SD). P-values are from Wilcoxon rank-sum tests. Abbreviations: CSF, cerebrospinal fluid; SAA, seed amplification assay; PD, Parkinson disease; MC1R, melanocortin 1 receptor; SD, standard deviation; NfL, neurofilament light chain; GFAP, glial fibrillary acidic protein; RFU, relative fluorescence units

eTable 10. SURE-PD phase 2, SURE-PD3, and STEADY-PD III demographics, clinical, and genetic characteristics

	SURE-PD phase 2		SURE-PD3	
	Non-carriers	*MC1R* LoF carriers	Non-carriers	*MC1R* LoF carriers
**Demographics**				
Participants	40	34	59	167
Age at PD onset (SD)	58.6 (10.4)	63.2 (10.1)	64.3 (7.1)	61.2 (10.3)
Male (%)	25 (62.5)	15 (44.1)	32 (54.2)	85 (50.9)
Race (%)				
Asian	0	0	1 (1.7)	0
Black	0	0	1 (1.7)	1 (0.6)

White	37 (92.5)	32 (94.1)	56 (94.9)	163 (97.6)
Unknown	3 (7.5)	2 (5.9)	1 (1.7)	3 (1.8)
**Clinical characteristics**				
Average follow-up (SD)	1.2 (0.6)	1.3 (0.7)	1.2 (0.9)	1.3 (0.9)
MDS-UPDRS III OFF score at baseline (SD)	21.1 (8.9)	22.9 (8.8)	20.9 (9.1)	21.9 (8.8)
MAO-B inhibitor use (%)	0	0	33 (55.9)	94 (56.3)
Active treatment arm (%)	23 (57.5)	26 (76.5)	35 (59.3)	84 (50.3)
**Genetic characteristics**				
MC1R LoF homozygotes (%)	----	0	----	9 (5.4)
LRRK2 carriers (%)	0	0	8 (13.6)	18 (10.8)
GBA carriers (%)	1 (2.5)	0	2 (3.4)	8 (4.8)

Statistical analysis comparing group characteristics was not performed. Abbreviations: PD, Parkinson disease; MC1R, melanocortin 1 receptor; SD, standard deviation; MDS-UPDRS III, Movement Disorder Society Unified Parkinson's Disease Rating Scale Part III; LRRK2, leucine-rich repeat kinase 2; GBA, glucocerebrosidase

eTable 11. Demographics for prodromal participants with and without *MC1R* variants

	Prodromal non-carrier	Prodromal *MC1R LoF* carrier
Participants	19	34
Enrolled for RBD (%)	13 (68.4)	21 (61.8)
Enrolled for hyposmia (%)	6 (31.6)	13 (38.2)
MC1R LoF homozygotes (%)	----	3 (8.8)
Age at baseline (SD)	69.0 (5.8)	69.4 (6.0)
Average follow-up (SD)	6.6 (3.0)	7.5 (3.3)
Male (%)	19 (100)	25 (73.5)
Race (%)		
Black	1 (5.3)	1 (2.9)
White	17 (89.5)	32 (94.1)
Unknown	1 (5.3)	1 (2.9)
History of melanoma	0	0
Phenoconverters		
PD	3 (15.8)	20 (58.8)
DLB	2 (10.5)	1 (2.9)
MSA	1 (5.3)	0

Statistical analysis comparing group characteristics was not performed. There were no participants with both RBD and hyposmia. Abbreviations: MC1R, melanocortin 1 receptor; PD, Parkinson disease; DLB, dementia with Lewy bodies; MSA, multiple system atrophy

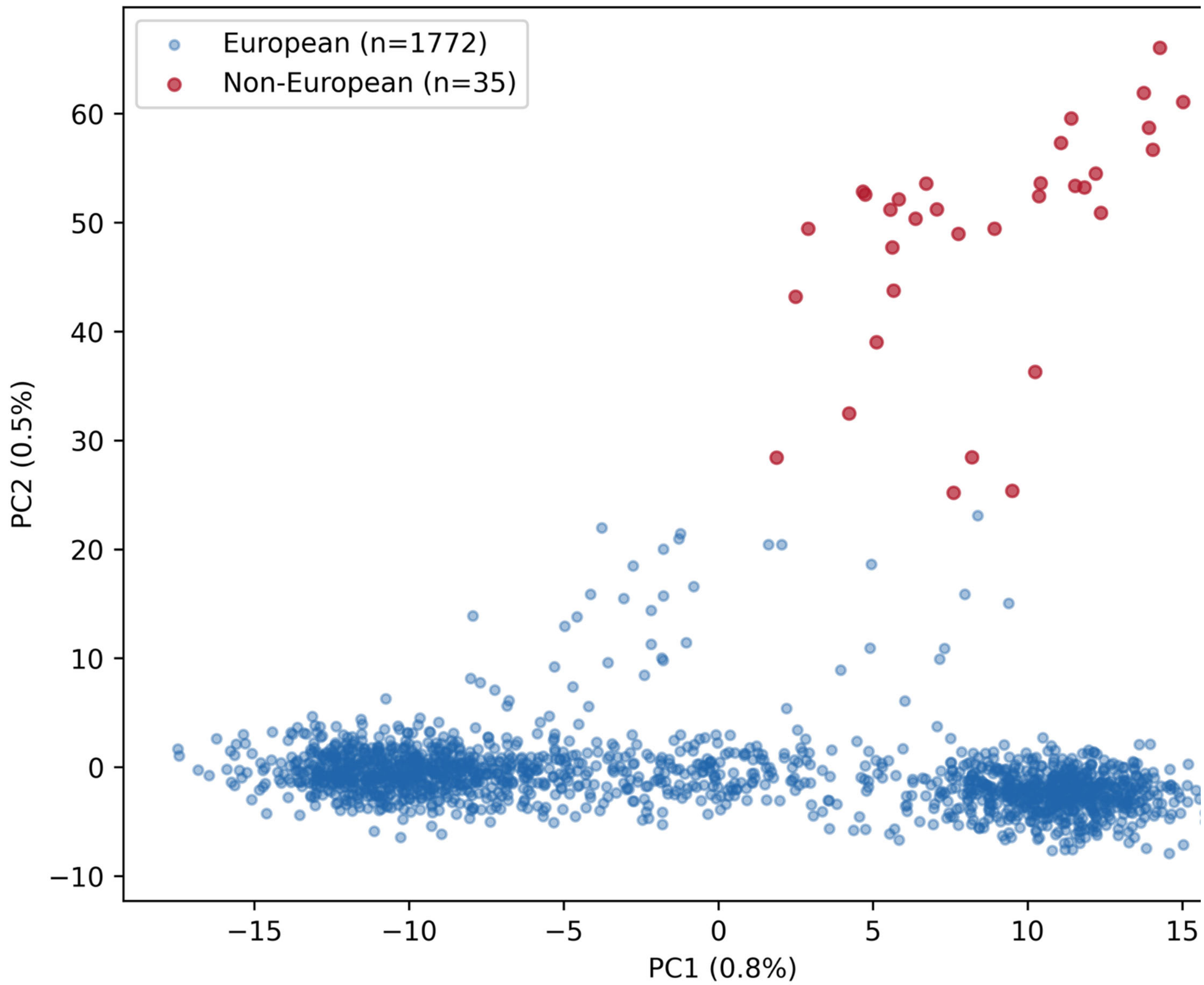

eFigure 1. Principal component analysis of PPMI participants

Projection of 1,807 PPMI participants onto the first two principal components computed from 4,924 LD-pruned common biallelic variants. Thirty-five participants (1.9%) were identified as non-European ancestry outliers (>3 SD on PC1 or PC2). PC1 explained 0.8% of genetic variance, indicating minimal population substructure within the cohort. Abbreviations: PC, principal component; SD, standard deviation

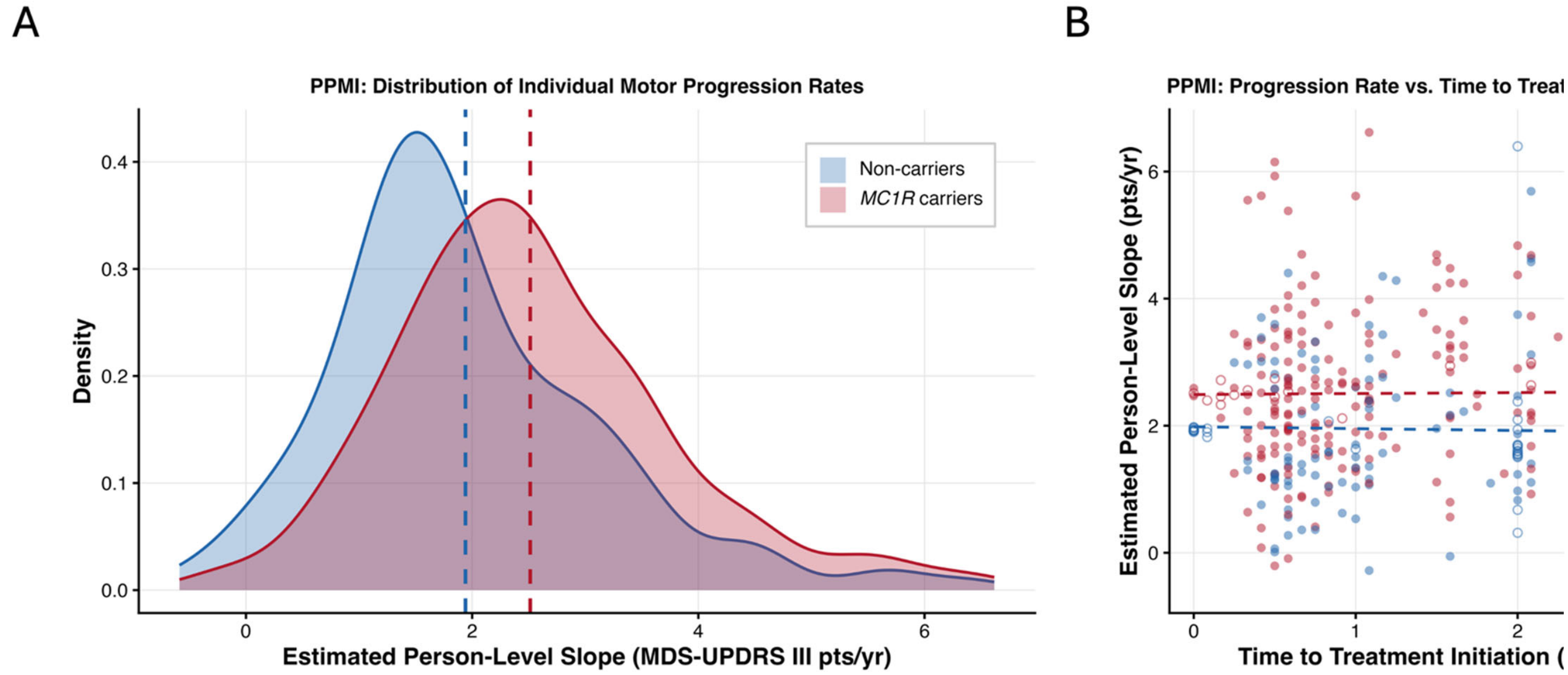

eFigure 2. Distribution of individual motor progression rates and relationship to treatment initiation in the PPMI cohort

(A) Distribution of estimated person-level motor progression rates in *MC1R* LoF carriers ("*MC1R* carriers") and non-carriers in the PPMI sporadic cohort. Person-level slopes were derived from random effects of the linear mixed-effects models. Dashed lines indicate group means. **(B)** Estimated person-level motor progression rate versus time to treatment initiation in the PPMI sporadic cohort. Filled points indicate participants who initiated levodopa or dopamine agonist therapy. Dashed lines represent linear trends within each group. Abbreviations: MC1R, melanocortin 1 receptor; MDS-UPDRS III, Movement Disorder Society Unified Parkinson's Disease Rating Scale Part III

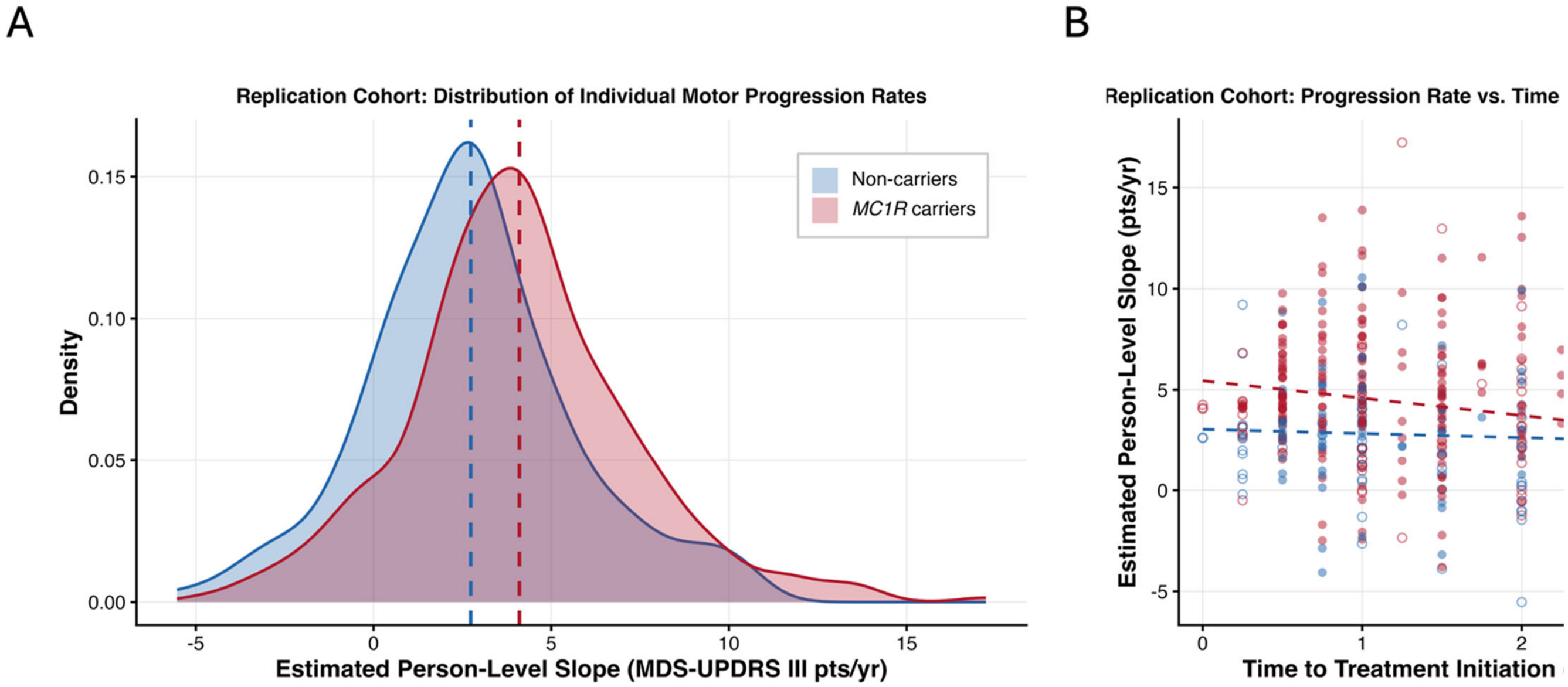

eFigure 3. Distribution of individual motor progression rates and relationship to treatment initiation in the replication cohort

(A) Distribution of estimated person-level motor progression rates in *MC1R* LoF carriers ("*MC1R* carriers") and non-carriers in the pooled replication cohort. Person-level slopes were derived from random effects of the linear mixed-effects models. Dashed lines indicate group means. **(B)** Estimated person-level motor progression rate versus time to treatment initiation in the pooled replication cohort. Filled points indicate participants who initiated levodopa or dopamine agonist therapy; open points indicate participants who remained untreated through the end of follow-up. Dashed lines represent linear trends within each group. The negative association between progression rate and time to treatment initiation illustrates survivorship bias: faster progressors initiate treatment earlier and are censored from later timepoints, causing observed means to underestimate population-level rates at longer follow-up. Abbreviations: MC1R, melanocortin 1 receptor; MDS-UPDRS III, Movement Disorder Society Unified Parkinson's Disease Rating Scale Part
