# Supplementary material for: Common MC1R Variants and Parkinson Disease Progression
Source: JAMA Neurol. 2026 Sep 8;83(10):981–9. doi: 10.1001/jamaneurol.2026.2998 (PMC13555505; doi:10.1001/jamaneurol.2026.2998)
Supplement: Supplementary file 2 [file jamaneurol-e262998-s002.pdf]

Data Sharing Statement

Schumacher. Common MC1R Variants and Parkinson Disease Progression. *JAMA Neurol*.
Published September 08, 2026. doi:10.1001/jamaneurol.2026.2998

Data
Data available: No

Additional Information
Explanation for why data not available: Data used in the preparation of this article are openly available to qualified researchers upon registering for access via PPMI and GP2. Data were obtained in February 2026 from the PPMI database (www.ppmi-info.org/access-data-specimens/download-data, RRID:SCR_006431) and March 2026 from the GP2 (https://gp2.org) database. A list of all data used, and the code generated in this study can be found at https://github.com/JacksonGS-1/MC1R_Progression_Code.
